# P-477. What Can be Learned from Vermonters Living with HIV Viremia?

**DOI:** 10.1093/ofid/ofae631.676

**Published:** 2025-01-29

**Authors:** Finlay Pilcher, Bradley Tompkins, Keara Lynn, Devika Singh

**Affiliations:** University of Vermont Medical Center, Burlington, Vermont; University of Vermont Medical Center, Burlington, Vermont; Larner College of Medicine at the University of Vermont, Burlington, Vermont; The University of Vermont Medical Center, Burlington, Vermont

## Abstract

**Background:**

Lower rates of viral suppression have been identified in people living with HIV (PLHIV) residing in rural compared to metropolitan areas. This is a barrier to ending the HIV epidemic in the US, and thus represents an urgent focus topic.

Vermont is a rural state with roughly 700 PLHIV. Vermonters living with HIV tend to be rigorously engaged into clinical care and have high rates of viral suppression. Yet, there are PLHIV in Vermont that have unsuppressed viral loads, and little is known about the characteristics of this cohort.

Our study aims to epidemiologically describe Vermonters with an HIV viral load greater than 200 copies/mL. This characterization can aid in appropriate allocation of resources and improve our understanding of PLHIV with viremia residing in rural areas.

**Figure:**
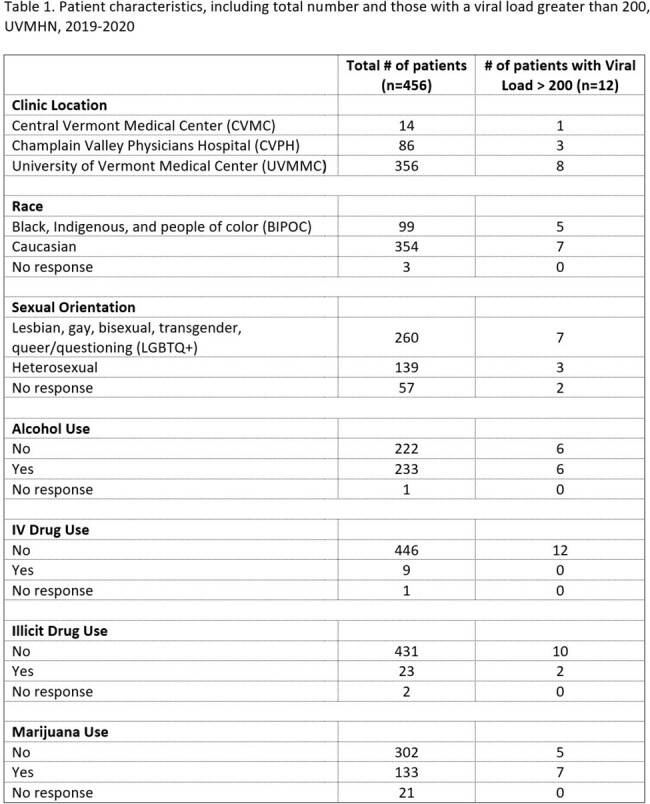

**Methods:**

Our study used electronic health record data from Epic Systems Corporation (Epic) from 2019 and 2020. We included PLHIV from three infectious disease clinic sites within the University of Vermont Health Network (UVMHN; n = 456) that had at least one clinical visit in the past 18 months. The following data were extracted from patient charts: HIV detection and quantitation, clinic location, race, sexual orientation, and substance use.

**Figure:**
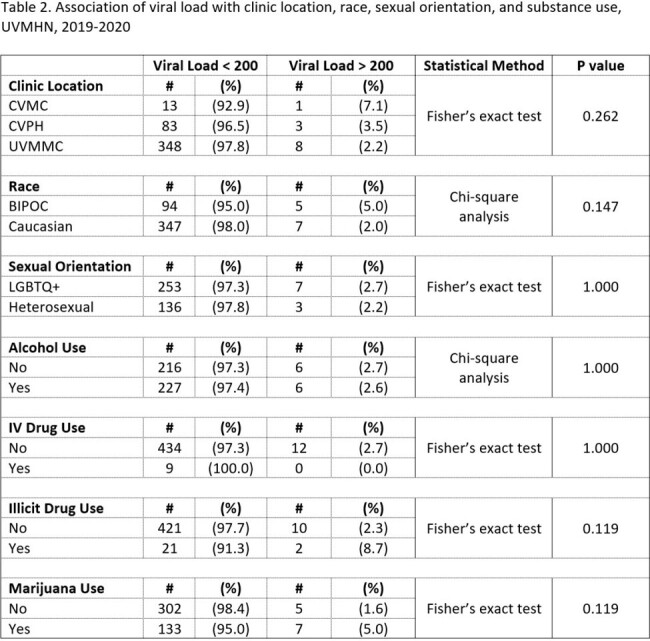

**Results:**

The results of our study are shown in Tables 1 and 2. There was no significant association between clinic location, race, sexual orientation, or substance use and having a viral load greater than 200 copies/mL.

**Conclusion:**

Our results suggest that in Vermont, the presence of viremia in PLHIV cannot be easily predicted by clinic location race, sexual orientation, or substance use. The authors suggest that traditional clinical markers including low CD4 counts, HIV resistance mutations, pill burden, older age and concomitant clinical comorbidities (dementia, etc.) may be more reliable factors associated with active viremia.

Although Vermont is demographically distinct from other rural areas, its residents share many barriers related to HIV care, including long travel distances to visits, limited HIV provider availability, and limited access to harm reduction services. These challenges necessitate our continued efforts to better understand and serve PLHIV living in rural areas to improve HIV equity and ultimately end the HIV epidemic.

**Disclosures:**

**All Authors**: No reported disclosures

